# Effect of Reclaimed Asphalt Pavement Heating Temperature on the Compactability of Recycled Hot Mix Asphalt

**DOI:** 10.3390/ma13163621

**Published:** 2020-08-16

**Authors:** Xiang Ma, Zhen Leng, Lili Wang, Peisheng Zhou

**Affiliations:** 1 College of Civil Engineering, Nanjing Forestry University, Nanjing 210037, China; m18752850095@163.com; 2 Department of Civil and Environmental Engineering, The Hong Kong Polytechnic University, Hong Kong; zhen.leng@polyu.edu.hk; 3 Suzhou Sanchuang Pavement Engineering Co., LTD, Suzhou 215128, China; mxfst@sina.com

**Keywords:** RAP, recycled hot mix asphalt, compactability, gyratory compaction, CER

## Abstract

The compactability of an asphalt mixture is related to the heating temperature of the materials, but the heating temperature of reclaimed asphalt pavement (RAP) is limited by the production process of hot-in-plant recycled mixtures. To choose a reasonable heating temperature for RAP according to the compactability, the compaction energy ratio (CER) obtained from the Superpave gyratory compactor compaction curve was developed. The CERs of fourteen kinds of asphalt mixtures made with different RAPs were compared, all of which were different in type, content, and heating temperature. The results indicated that CER is an effective energy index to evaluate the workability of a bituminous mixture, and it considers both the accumulated energy after each gyration and the number of gyrations. It was also found that increasing the heating temperature of the RAP cannot always improve the workability of the recycled mixture, because the higher heating temperature caused more hard-aged bitumen to be blended with soft virgin bitumen during the mixing process. At the same RAP heating temperature, increasing the RAP content made it more difficult to compact the mixture, especially for RAPs with styrene–butadiene–styrene (SBS) modified bitumen, and the recycled mixtures with SBS-modified bitumen were more difficult to compact than those with nonmodified bitumen.

## 1. Introduction

With the increasing demand for asphalt pavement rehabilitation, an increasing amount of reclaimed asphalt pavements (RAPs) are being produced and need to be recycled. As RAP contains valuable asphalt binders and aggregates, they can replace the virgin binder, and can replace aggregate in new asphalt pavement construction and asphalt pavement rehabilitation, providing significant environmental and economic benefits [1]. The recycling techniques are a hot research topic in the field of road engineering [2,3,4]. According to the recycling processes and mixing temperature, the recycling methodologies can be classified as hot recycling (HR) and cold recycling (CR) [5]. Although CR possesses superior advantages in its environmental effect [6], the HR recycling technique has a wide range of applications because of its improved performance [7].

As with fresh hot mix asphalt (HMA), temperature is considered a key factor affecting the compactability of recycled HMA, and poorly compacted mixtures may lead to severe rutting, poor fatigue resistance, short durability, and premature moisture damage [8,9]. Increasing the heating temperature of RAP is typically performed in a drum mix plant so as to increase the temperature of recycled RAP. 

However, such a temperature increase does not always correspond to increased compactability of the recycled mixture. Many studies have reported that the aged binder in RAP and virgin binder are be blended at some degree of 0% (black rock) to 100% (full blending) [10,11,12,13,14]. In the zero-blending condition, the RAP binder acts like a rock, in that it does not coat other virgin aggregates, and if there is full blending, the properties of the binder around the virgin aggregates will be similar to that of the binder around the RAP. The mixing temperature plays a significant role in increasing the blend ratio, because the hot virgin aggregates and the hot virgin binder can assist in melting the RAP binder, allowing for further diffusion of the two binders into one another [15,16]; at the same time, the penetration of the rejuvenator accelerates the blending between the virgin and aged asphalt binder [17,18]. Therefore, the heating temperature of RAP will surely affect the degree of blending of the two binders. The higher the heating temperature, the higher the degree of blending that can be achieved because of the more active aged binder. However, the effects of increasing the RAP heating temperature are two-fold. On the one hand, a more active aged binder may introduce a more aged binder into the mixed binder, causing a viscosity increase. On the other hand, the viscosity of the mixed binder will be decreased as a result of the temperature increase. Therefore, the effect of the RAP heating temperature on the compactability of the recycled HMA is very complicated, which is a challenge of the two aforementioned effects.

The main objective of this study is to investigate the compactability of recycled HMAs prepared with different RAP heating temperatures. To achieve this objective, two common RAP materials used in China were selected, namely: (1) a surface layer mixture, AC-13, with SBS modified bitumen and a basalt aggregate and (2) a middle/lower layer mixture, AC-20, with nonmodified bitumen and a limestone aggregate. In addition, three RAP percentages (0%, 20%, and 40%) and three RAP heating temperatures during mixture production (100, 120, and 140 °C) were considered. A new index, the compaction energy ratio (CER), obtained from the Superpave gyratory compactor compaction curve, was developed to quantify the compactability of different mixtures.

## 2. Materials and Experimental Program

### 2.1. Materials

To achieve a more systematic evaluation, two typical RAP materials used in China were selected in this study. The first one is the AC-13, which is a surface layer mixture with a nominal maximum aggregate size (NMAS) of 13.2 mm. It was made with SBS modified bitumen and basalt aggregate. The second one is an AC-20 mixture, which is a middle/lower layer mixture with an NMAS of 19.0 mm. It was made with nonmodified base bitumen and limestone aggregate.

Two types of recycled HMA were produced from the two RAPs, namely: recycled AC-13 and recycled AC-20. The recycled AC-13 was made with AC-13 RAP, SBS modified bitumen, basalt aggregate, and mineral filler, while the recycled AC-20 was made with AC-20 RAP, base bitumen, limestone aggregate, and mineral filler. The recycled HMAs were produced at three RAP percentages (0, 20%, and 40%) and three RAP heating temperatures (100 °C, 120 °C, and 140 °C). 

Based on the aforementioned testing variables, 14 HMA mixtures were prepared in a laboratory, as shown in Table 1. The recycled mixture was given a coded name according to the NMAS, percentage of RAP (%), and the RAP heating temperature (°C), for example, A-B-C, where A represents NMAS, B represents percentage of RAP (%), and C represents RAP heating temperature (°C).

### 2.2. RAP Characterization

- Binder content

Among the most important properties of asphalt mixtures is the binder content. In order to determine the binder content of RAP, extraction tests were conducted using a centrifuge with trichloroethylene as the solvent, in accordance with Chinese specification T 0722-1933 [19].

- Gradation analysis

After the binder extraction tests, the remaining mineral mixture was placed in an oven at 105 °C to dry. After drying, the mineral mixture was cooled to room temperature and sieve analysis was conducted to determine the aggregate gradation of RAP.

- Binder recovery and characterization

To evaluate the aged binder in the RAPs, RAP samples were first dissolved in a toluene solvent to remove all solid particles with filter and centrifuge. Then, the RAP binder was recovered by vacuum distillation using a rotary evaporator, according to the Chinese specification T 0727-2011 [19]. Finally, the recovered binder was characterized through penetration (T 0604-2011), ductility (T 0605-2011), and softening point tests (T 0606-2011) [19].

### 2.3. Recycled HMA Mix Design

- Gradation design

The recycled HMA with the same NMAS and the same target gradation was designed according to the Chinese specification JTG F40-2004 [20]. In the gradation design of recycled HMA, the aged aggregate was seen as a kind of aggregate based on the particle size distribution. We fixed the percentage of aged aggregate to be the same as the percentage of RAP and adjusted the proportion of new aggregate to make the synthetic gradation of the aged and virgin aggregate to be close to the target gradation.

- Optimum binder content

The optimum binder content of the recycled HMA was determined by the Marshall method (75 blows per side), according to the Chinese specification JTGT 5521-2019 [21]. In the process of mix design, the heating temperature of the RAP was 120 °C.

### 2.4. Compactability Evaluation

Superpave gyratory compaction is considered to be one of the best methods to assess the compactability of asphalt mixtures [22,23,24]. For this reason, a gyratory compactor was used to investigate the compaction characteristics of different recycled mixtures. The method of preparation of the specimens used the following procedure:

1. The RAPs were put into an oven at the needed temperature to achieve the desired heat; the heating time was not more than 2 h so as to avoid the RAP being further aged.
2. The heating temperature of the virgin aggregate was higher than the mixing temperature of 10–15 °C; the limestone was heated to 175 °C and the basalt was heated to 165 °C.
3. The order of addition of the materials was firstly a virgin aggregate and RAP mixed in the mixer for 1 min; secondly, the new binder was added; and, finally, the heated filler was added. Then, all of the materials were mixed until homogeneous, and the total mixing time was generally 3 min.
4. After the mixing process, the specimens were compacted using the Superpave gyratory compacter.

A total of 160 gyrations were applied to all of the mixtures, with a compaction pressure of 600 kPa and a compaction angle of 1.25°. During the gyratory compaction, the relationship between the number of gyrations and the height of the specimen was recorded, which is referred to as the compaction curve. Based on the compaction curve, the bulk volume density of the specimen after each gyration could be estimated from its height. Then, the degree of compaction (DC) could be calculated, which was equal to the percentage of bulk volume density to the theoretical maximum density of the compacted mixture. The air void content (*V*) and DC held the following relation:

(1) $$V_{i}=100-DC_{i}$$

where *V_i_* is the air void content for a given number of gyration (%), and *DC_i_* is the degree of compaction for a given number of gyration (%).

- Compaction energy index

**Figure 1:**
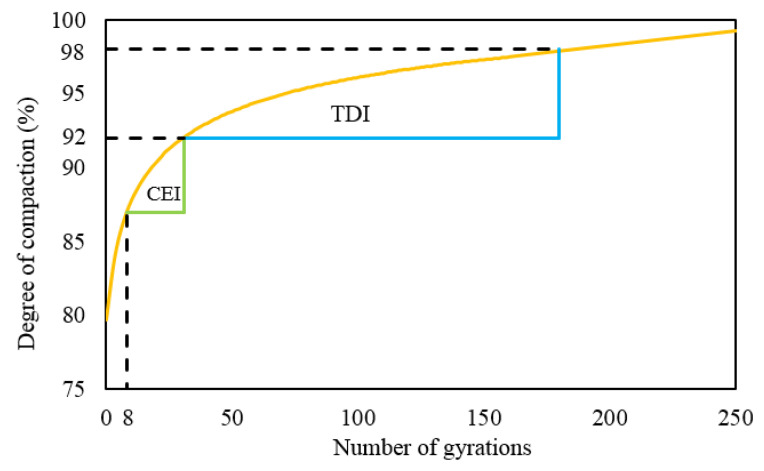
Schematic illustration of the definition of compaction energy index (CEI) and traffic densification index (TDI).

In the literature, many of the indices obtained from the SGC compaction curves have been reported and used to evaluate the asphalt mixture compactability, and the compaction energy index (CEI) and traffic densification index (TDI) were used generally [23,25], which were determined using the compaction curve data (as illustrated in Figure 1) for measuring compaction energy [26,27]. The CEI index focused on the results from cycle 8 until a DC of 92% was reached, which corresponded to the minimum density for traffic opening [28]. The TDI index was related to the possible postdensification as a result of the traffic effects. It was determined from the data within the DC range of 92% to 98% [29]. Within this range, the mixture approached the plastic behavior zone.

The CEI value indicates the ease of laying mixes during construction, which is used to evaluate the compactability of HMA. The lower this value, the more easily the mixture can be compacted. 

As indicated, the relation between the accumulated compaction energy and the number of gyrations follows a simple linear relation. As a result, Elsa [23] proposed a linear equation to fit the data, namely, Equation (2):

(2) $$A_{A}=aN_{i}-b$$

where *A_A_* is the accumulated area, and *a* and *b* are the regression coefficients.

- Compaction energy ratio

**Figure 2:**
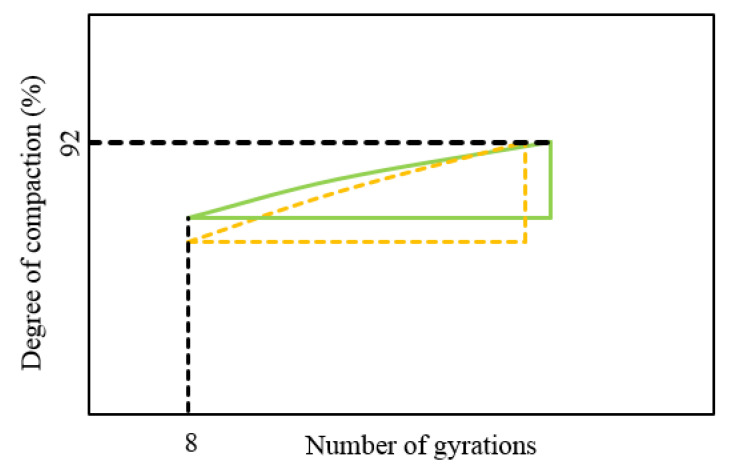
Different curves with an equal CEI value.

It is worth noting that the CEI cannot directly be used to determine the change of compaction energy in the process, and the number of gyrations to reach a DC of 92% has a significant effect on the value of CEI. For example, in Figure 2, the values of CEI are equal for the two curves, but it is obvious that the mixture represented by the lower curve has a better compactability, because its DC was higher after the same number of gyrations at this stage.

**Figure 3:**
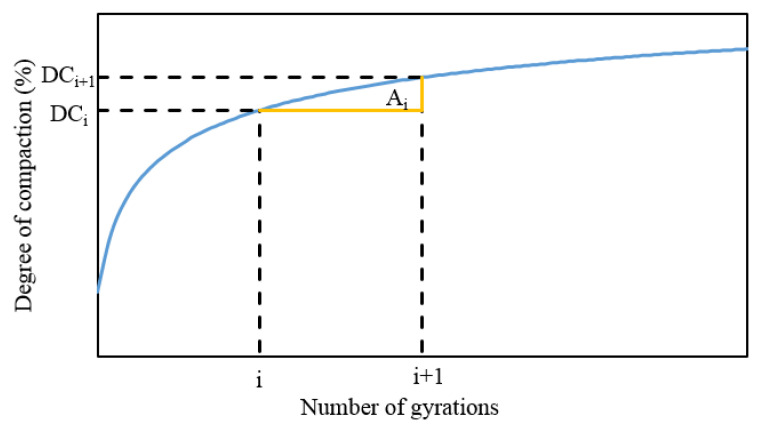
The area of the adjacent number of gyrations under the compaction curve.

In this study, the energy index was modified to consider the effect of each cycle of gyration on the compaction characteristics [30]. As illustrated in Figure 3, the area of the adjacent numbers of gyration, Ai, could be calculated. This area reflects the change of DC after one gyration cycle. The larger the area, the greater the change of DC, and the HMA is more easily compactable.

Under this curve, the accumulated area at N cycles of gyration ($A_{A_{N}}$) can be calculated as follows:

(3) $$A_{A_{N}}=\sum_{i=1}^{i=N-1}A_{i}=\sum_{i=1}^{i=N-1}\frac{DC_{i+1}-DC_{i}}{2}$$

**Figure 4:**
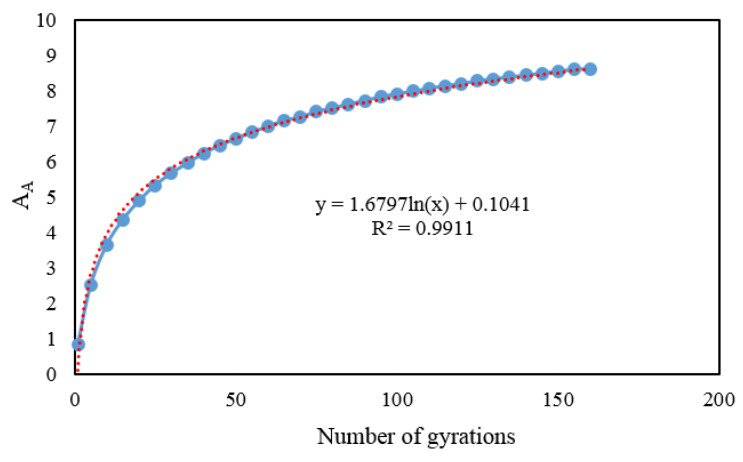
Relation between the accumulated area and the number of gyrations.

Figure 4 shows the relation between the accumulated area (*A_A_*) and number of gyrations. A regression equation can be obtained through a logarithmic fitting using Equation (4).

(4) $$A_{A_{N}}=aLn\left(N\right)+b$$

where *N* is the number of gyrations, $A_{A_{N}}$ is the accumulated area after *N* cycles of gyration, and *a* and *b* are the regression coefficients.

As the area at the given adjacent number of gyrations can only reflect the compaction characteristics at this point, the accumulated area and the number at this stage should be considered. Therefore, in the study, the compaction energy ratio (CER) was developed to evaluate the compaction characteristics of HMA. Assuming *A* and *B* represent different number of gyrations, and *B* is larger than *A*, the following equation can be used to calculate the CER between *A* and *B* (${\mathrm{CER}}_{A\;to\;B}$):

(5) $${\mathrm{CER}}_{A\;to\;B}=\frac{A_{A_{B}}-A_{A_{A}}}{B-A}$$

where $A_{A_{A}}$ is the accumulated area at *A* cycles, and $A_{A_{B}}$ is the accumulated area at *B* cycles.

When the number of gyrations increases from A to B, ${\mathrm{CER}}_{A\;to\;B}$ can reflect the average change of the compaction degree after one cycle, and the value is not affected by the DC at A cycles.

## 3. Results and Discussions

### 3.1. Virgin Material Properties 

Table 2 and Table 3 present the properties of the virgin binder and the two types of aggregate used in this study. It can be observed in Table 2 and Table 3 that the modified bitumen had less penetration than the base bitumen, a higher softening point and higher viscosity, and basalt had a larger density and strength than limestone.

### 3.2. RAP Characterization

As Table 4 shows, the binder contents for AC-13 RAP and AC-20 RAP were 4.7%, and 4.0%, respectively, and the gradations of both RAPs were obviously finer than the recommended gradation of the Chinese specification, which was expected as a result of the breakage of aggregates during the milling process. 

As Table 5 shows, for both the modified binder and base binder, the recovered bitumen was stiffer than the virgin bitumen (Table 2), as a result of aging.

### 3.3. Recycled HMA Design

**Figure 5:**
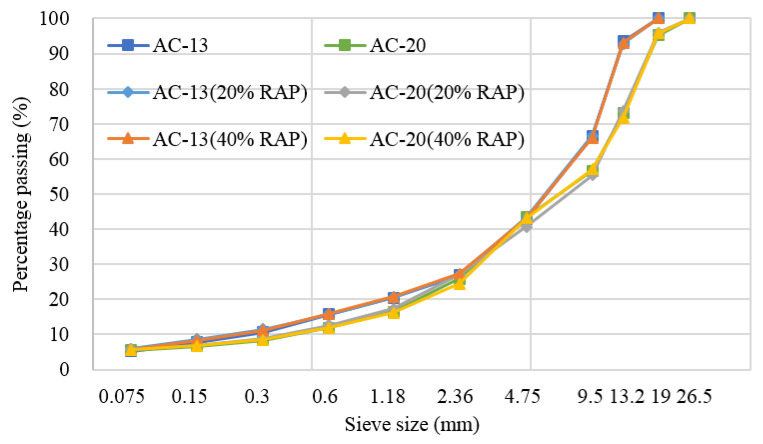
Gradation of different asphalt mixtures.

Figure 5 shows the gradation of both the virgin and recycled HMA. It can be seen that the gradations of the recycled HMA were very close to the virgin HMA. Therefore, the influence of gradation on the compaction characteristics can be weakened. 

According to JTG F40-2004 [20], the optimum bitumen content (OAC) can be calculated based on the relationship between the bitumen content and the Marshall test indexes, including bulk density, air void, voids in the mineral aggregate (VMA), voids filled with asphalt (VFA), Marshall stability, and Marshall flow. Table 6 presents the Marshall mix design results of all of the HMAs prepared in this study, and OAC is the quality ratio of virgin asphalt to mixture. It can be seen that the actual OAC of the recycled HMA was slightly larger than the predicted asphalt content (*P_nb_*), probably because some aged bitumen was inactive. This phenomenon is in agreement with some previous studies’ findings [31,32,33]. Furthermore, the stability of the virgin HMA was lower than those of the recycled HMA, and the difference was more significant at a higher RAP percentage. The Marshall quotient is the ratio of Marshall stability over the flow value; RAP can increase the quotient of HMA, which means the use of RAP can improve the stiffness of the HMA.

### 3.4. Compactability of Recycled HMA

An air void content of 8% is usually regarded as the maximum allowable air void content for the compaction quality control of asphalt pavements. With respect to mixture design, a void content of 4% is usually desired. Therefore, in this study, two sections were considered when analyzing the compactability: one section was the section from N = 8 to V = 8%, and the other section was from N = 8 to V = 4%.

In order to calculate the values of CEI and CER for a given section, the corresponding number of gyrations should be known. As in other studies [24,26], the following equation can be used to explain the relation between air void and the number of gyrations:

(6) $$V_{i}=V_{1}-KLn\left(N_{i}\right)$$

where *V_i_* is the air void content for a given number of cycles (%), *V*_1_ is the air voids content calculated at the first gyration, and *K* is the compactability factor. *N_i_* is the number of gyrations.

The corresponding number of gyrations can be calculated using Equation (6), and the regression results are listed in Table 7.

The regression coefficients of *K* and *V*_1_ were used to explain the ease of compaction, but *V*_1_ is mainly related to the initial accumulation state of the mixture, and *K* can only be used to compare mixes if they have the same *V*_1_. Therefore, when using the indices of *K* and *V*_1_ to compare the compactability of different asphalt mixtures, the mixtures should have the same initial accumulation state. As can be seen from Table 7, the virgin mixture did not have the highest *K* or the lowest *V*_1_, which contradicts common sense. It might be because the virgin mixture had a different initial accumulation state than the recycled HMA.

For AC-20 HMA, the virgin mixture required more gyration cycles to reach the 8% air void content than the recycled mixture with 20% RAP heated to 140 °C, which contradicts common sense, as described above. The reason is that during the early compaction stage, the contrast relationship between the void content was greatly affected by *V*_1_, and the virgin mixture had a higher *V*_1_ than the recycled mixture with 20% RAP heated to 140 °C. As the number of gyrations increased, the effect of *V*_1_ decreased, and the contrast in the relationship was greatly affected by the compactability; therefore, it can be seen that the virgin mixture required less gyration cycles to reach the 4% air void content than the recycled mixture with 20% RAP heated to 140 °C, which contradicts common sense, as described above. Therefore, the number of gyrations to reach the target void content is not always a good index to evaluate the compactability of HMA, because it is difficult to determine when the effect of *V*_1_ becomes smaller.

Fitting gyratory compaction data using Equation (2) led to the regression results listed in Table 8. CEI_1_ is the accumulated compaction energy from N = 8 to V = 8%, and CEI_2_ is the accumulated compaction energy from N = 8 to V = 4%.

It is obvious from Equation (2) that the number of gyrations affected the accumulated compaction energy, but the number of gyrations to reach the target void content was affected by *V*_1_. As can be seen from Table 5, the virgin mixture did not always have the smallest CEI_1_ and CEI_2_. For the same NMAS mixture, the CEI_1_ and CEI_2_ had a different order between the different recycled HMAs. Therefore, CEI is also not a good index to evaluate the compactability of HMA.

CER as an effective and better index was developed to evaluate the compactability of HMA. Fitting gyratory compaction data using Equation (4) led to the regression results listed in Table 9. CER_1_ is the compaction energy ratio from N = 8 to V = 8%, and CER_2_ is the compaction energy ratio from N = 8 to V = 4%.

The following can be observed from Table 9:

1. For both the HMA with SBS modified bitumen and the HMA with a base bitumen, the virgin HMA had the largest CER in the roller-compacted stage, indicating that the virgin HMA was the easiest to compact, which is to be expected. For the same NMAS mixture, CEI_1_ and CEI_2_ had the same order between the different recycled HMAs. CER is an effective index to evaluate the compactability of HMA, because it considers both the number of gyrations and the accumulated compaction energy to reach a target void content.
2. For the recycled AC-20 mixture with 40% RAP, a higher heating temperature of RAP led to a larger CER. However, for both the AC-13 mixture and AC-20 mixture, out of the three types of recycled HMAs containing 20% RAP, the recycled HMA with RAP heated to 120 °C had the largest CER, and for the recycled AC-13 mixture with 40% RAP, the recycled HMA with RAP heated to 120 °C had almost the same CER as the recycled HMA with RAP heated to 140 °C. It could be expected that it is not enough to simply increase the heating temperature of RAP to improve the compactability of recycled HMA. It is possible that when the heating temperature of RAP is increased, more aged bitumen is blended with the virgin bitumen during the mixing process, which overcomes the effect of viscosity decrease due to a temperature increase.
3. For the same NMAS and same RAP percentage, the value of CER for the recycled HMA with RAP heated to 100 °C was the smallest, indicating that 100 °C was too low for RAP heating.
4. For the same NMAS and same RAP heating temperature, with the increase in RAP percentage, the value of CER decreased.

Based on the above analysis, to improve compactability, RAP should be heated at a suitable temperature, depending on the type and percentage of RAP. It is not always true that a higher heating temperature of RAP will lead to easier compaction. For the RAPs evaluated in this study, the recycled HMA containing 20% RAP heated to 120 °C had, in general, better compactability than that heated to 140 °C.

## 4. Conclusions

In the study, the compaction characteristic of different recycled HMAs were investigated and compared through laboratory testing. Based on the outcome of the experimental study, the following conclusions can be drawn:

1. CER is a new energy index, which considers both the accumulated energy after each gyration and the number of gyrations. It is a better index compared to CEI, for the purpose of evaluating the compactability of HMA.
2. Increasing the heating temperature of RAP is not always an effective method to improve the compactability of recycled HMA. In the study, the recycled HMA mixtures containing 20% RAP are easier to compact with RAP heated to 120 °C than the same mixtures with RAP heated to 140 °C.
3. Higher RAP contents make the recycled HMA more difficult to compact, especially for RAP with SBS modified bitumen.
4. The recycled HMA with SBS modified bitumen is more difficult to compact compared with those with base bitumen.
5. An optimum RAP heating temperature exists for producing recycled HMA with the best compactability, depending on RAP type and RAP content.

Based on the above findings, the authors consider CER to be a more reasonable index than CEI as a compactalibity parameter of recycled hot mix asphalt. However, further research is required to evaluate the compactalibity of other kinds of HMA, and additional types of binder and gradation should be included to verify the advantage of CER.

## Figures and Tables

**Table 1:** HMA mixtures prepared for compaction characterization.

Mixture Type	NMAS (mm)	Bitumen Type	AGGREGATE Type	Percentage of RAP (%)	RAP heating Temperature (°C)
AC-20	19.0	base bitumen	limestone	0	—
20-20-100	19.0	base bitumen	limestone	20	100
20-20-120	19.0	base bitumen	limestone	20	120
20-20-140	19.0	base bitumen	limestone	20	140
20-40-100	19.0	base bitumen	limestone	40	100
20-40-120	19.0	base bitumen	limestone	40	120
20-40-140	19.0	base bitumen	limestone	40	140
AC-13	13.2	SBS modified bitumen	basalt	0	—
13-20-100	13.2	SBS modified bitumen	basalt	20	100
13-20-120	13.2	SBS modified bitumen	basalt	20	120
13-20-140	13.2	SBS modified bitumen	basalt	20	140
13-40-100	13.2	SBS modified bitumen	basalt	40	100
13-40-120	13.2	SBS modified bitumen	basalt	40	120
13-40-140	13.2	SBS modified bitumen	basalt	40	140

**Table 2:** Properties of virgin bitumen.

Test Item	Test Result		Test Method
	SBS Modified Bitumen	Base Bitumen	
Penetration (25 °C, 100 g, 5 s; 0.1 mm)	53	68	T0604-2011
Ductility (5 cm/min; mm)	32 (5 °C)	>100 (15 °C)	T0605-2011
Softening point (°C)	78	46.5	T0606-2011
Viscosity at 135 °C (Pa.s)	2.35	0.36	T0625-2011

**Table 3:** Properties of the virgin aggregates.

Aggregate Type	Test Item	Test Result		Test Method
		Basalt	Limestone	
Coarse aggregate	Apparent specific gravity	2.934	2.763	T0304-2005
	LA abrasion (%)	12.5	14.2	T0312-2005
	Crush value (%)	11.4	17.3	T0316-2005
	Absorption (%)	1.13	0.89	T0307-2005
Fine aggregate	Apparent specific gravity	2.853	2.748	T0328-2005
	Sand equivalent value	71	64	T0334-2005

**Table 4:** RAP gradation and bitumen content (after extraction).

RAP Type	AC-20	AC-13
Bitumen content (% by weight of mixture)	4.0	4.7
Sieve size (mm)	Gradation (% passing)	
26.5	100	100
19	97.3	100
13.2	73.4	94.1
9.5	60.7	79.5
4.75	42.6	49.5
2.36	23.7	34.2
1.18	16.3	23.8
0.6	11.7	16.3
0.3	6.9	13.1
0.15	5.1	9.7
0.075	3.9	6.1

**Table 5:** Properties of bitumen recovered from the RAP.

Test Item	Test Results		Test Method
	RAP (AC-13)	RAP (AC-20)	
Penetration (25 °C, 100 g, 5 s) (0.1 mm)	23	26	T0604-2011
Ductility (5 cm/min, 15 °C) (mm)	23.6	10.7	T0605-2011
Softening point (°C)	80.5	58.5	T0606-2011

**Table 6:** Marshall mix design results.

Mixture Type	*P_nb_* (%)	OAC (%)	Air Voids (%)	VMA (%)	Stability (kN)	Flow (mm)	Quotient (kN/mm)
AC-13	5.0	4.9	4.1	14.2	12.2	3.2	3.8
AC-13 (20% RAP)	4.1	4.2	4.2	14.4	13.4	3.2	4.2
AC-13 (40% RAP)	3.1	3.3	4.0	14.2	15.6	3.5	4.5
AC-20	4.2	4.1	4.2	13.1	11.6	3.2	3.6
AC-20 (20% RAP)	3.4	3.4	4.4	13.2	11.9	3.3	3.6
AC-20 (40% RAP)	2.6	2.7	4.2	13.3	15.3	3.7	4.1

**Table 7:** Regression results of Equation (6) and *N.*

Mixture Type	*K*	*V*_1_	*R* ^2^	*N_V=8%_*	*N_V=4%_*
AC-20	3.694	20.8	0.998	32	95
20-20-100	3.659	21.1	0.998	35	106
20-20-120	3.813	21.6	0.997	35	100
20-20-140	3.480	20.0	0.997	31	99
20-40-100	3.640	21.6	0.997	41	124
20-40-120	3.799	21.9	0.997	39	111
20-40-140	3.567	20.5	0.998	33	103
AC-13	3.486	20.0	0.998	31	98
13-20-100	3.424	20.3	0.999	37	118
13-20-120	3.580	20.6	0.998	34	104
13-20-140	3.564	20.7	0.998	35	108
13-40-100	3.378	20.7	0.996	43	141
13-40-120	3.295	19.8	0.987	36	120
13-40-140	3.455	20.7	0.997	39	125

**Table 8:** Regression results of Equation (2) and the CEI.

Mixture Type	*a*	*b*	*R* ^2^	CEI_1_	CEI_2_
AC-20	14.48	188.9	0.993	156	1265
20-20-100	14.26	186.9	0.993	187	1343
20-20-120	15.51	195.5	0.994	193	1285
20-20-140	13.62	178.5	0.993	137	1159
20-40-100	14.68	186.7	0.994	268	1737
20-40-120	15.45	194.8	0.994	242	1504
20-40-140	14.08	182.5	0.993	163	1265
AC-13	14.21	176.3	0.994	143	1152
13-20-100	13.69	174.2	0.994	210	1480
13-20-120	14.16	182.6	0.993	177	1292
13-20-140	14.13	181.8	0.994	187	1364
13-40-100	14.04	172.6	0.995	282	1958
13-40-120	14.21	169.3	0.995	204	1567
13-40-140	13.82	176.8	0.994	230	1664

**Table 9:** Regression results of Equation (4) and the compaction energy ratio (CER).

Mixture Type	*a*	*b*	*R* ^2^	CER_1_	CER_2_
AC-20	1.757	−0.2277	0.995	1009	1310
20-20-100	1.742	−0.2689	0.996	944	1215
20-20-120	1.807	0.0556	0.995	986	1277
20-20-140	1.656l	−0.2337	0.995	969	1250
20-40-100	1.728	−0.0203	0.995	850	1080
20-40-120	1.800	0.0554	0.995	926	1189
20-40-140	1.696	0.1723	0.996	953	1228
AC-13	1.652	0.0896	0.997	968	1250
13-20-100	1.625	−0.0636	0.997	864	1098
13-20-120	1.701	−0.1496	0.996	947	1219
13-20-140	1.694	−0.1368	0.996	926	1188
13-40-100	1.598	0.2064	0.996	766	959
13-40-120	1.555	0.4477	0.999	840	1064
13-40-140	1.641	−0.0748	0.996	837	1059
